# Bioengineered Protein Stabilized Perovskite Nanoplates in Polar Solvents

**DOI:** 10.1021/acs.nanolett.6c00282

**Published:** 2026-03-08

**Authors:** Emma H. Massasa, Oren Bachar, Arad Lang, Omer Yehezkeli, Yehonadav Bekenstein

**Affiliations:** † Department of Materials Science and Engineering, Technion − Israel Institute of Technology, 3200003 Haifa, Israel; ‡ Faculty of Biotechnology and Food Engineering, Technion − Israel Institute of Technology, 3200003 Haifa, Israel; § The Resnick Sustainability Center for Catalysis, Technion − Israel Institute of Technology, 3200003 Haifa, Israel; ∥ The Solid-State Institute, Technion − Israel Institute of Technology, 3200003 Haifa, Israel

**Keywords:** Lead halide perovskites, Nanomaterials, Protein-templated nanocrystals, Bioengineered protein scaffolds, Stability, Colloidal synthesis, Biotic-abiotic interface

## Abstract

Colloidally stabilized halide perovskite nanoparticles are promising for photocatalysis due to high absorption cross-section, high photoluminescence quantum yield, and nanoscale dimensions comparable to molecular reactants. Yet, their ionic lattice limits stability in polar media, complicating integration into aqueous catalytic systems. Here, we utilize stable protein 1 (SP1) as a bioinspired capping layer to protect perovskite nanoparticles from polar degradation while preserving their optoelectronic functionality, which stems from SP1’s amphiphilic nature, featuring a hydrophilic exterior and a hydrophobic interior. Using bioengineered SP1 and its monomeric derivatives, we synthesize perovskite nanoplates with tunable quantum confinement that remain stable in an isopropanol polar environment. Protein screening reveals that stability in organic solvents, combined with suitable surface amine and carboxylate distributions, is crucial for nanoplate formation and stability, resulting in hybrids with superior spectroscopic stability in polar media. These findings provide design rules for protein-stabilized perovskites, paving the way for future solution-phase biocatalytic systems.

## Graphical abstract

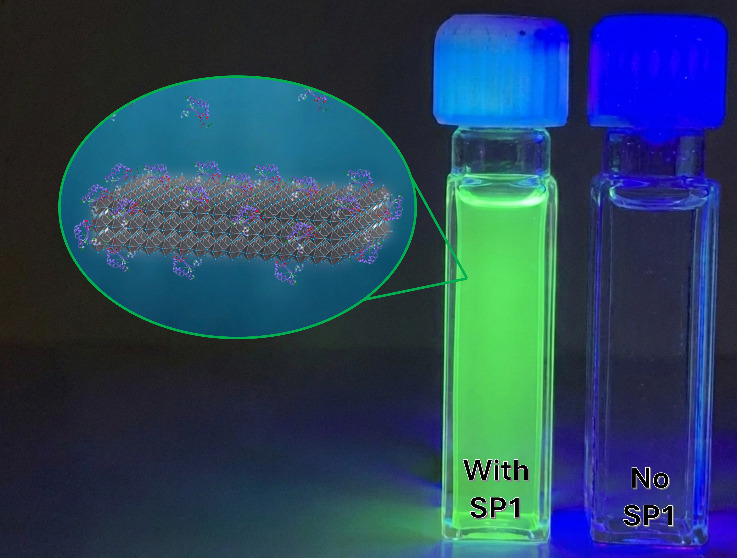

Lead halide perovskite (LHP) nanoparticles, with an ABX_3_ structure where A is a monovalent cation (Cs^+^), B is a divalent cation (Pb^2+^), and X is a halide anion (Cl^–^, Br^–^ or I^–^), exhibit great potential for optoelectronic applications due to their narrow emission spectra and high photoluminescence quantum yield (PLQY).^1–5^ LHPs are used in applications such as solar cells^6–9^ and LEDs.^5,10–14^ However, their integration into practical applications has been limited by poor stability, as they degrade in ambient conditions, particularly in the presence of humidity.^3,15,16^ Photocatalysis is a field in which LHP nanoparticles can contribute due to their large absorption cross-section and dimensions that are similar to those of target molecules.^17–23^ Also, many catalytic systems operate in aqueous environments, either due to functional requirements or to meet environmental sustainability goals, making it challenging to harness the advantages of perovskite nanoparticles in this context.

The most common ligands used to stabilize the colloidal LHP nanoparticles are oleyl amine (OLA) and oleic acid (OA).^24–26^ Other ligands, such as longer/shorter chain amines and phosphonic cations, as the functional group, are also common.^27–32^ In addition, zwitterionic ligands bearing both positively and negatively charged functional groups can bind simultaneously to different surface sites on the perovskite, yielding nanoparticles with improved chemical stability.^33–35^ However, the conventional ligands for LHP nanoparticles are only loosely bound to the surface. NMR studies demonstrated a reversible dynamic process of ligands that constantly attach to and detach from the surface.^36^ This can be problematic when the particles are dispersed in polar solvents, where ligand detachment is more likely, leading to surface defect formation and colloidal instability. Moreover, amino acids and peptides are used as stabilizing ligands.^37–41^ For example, Prochazkova et al. reported spherical particles with good stability due to the self-organization of amino acids on the nanoparticle surface.^37^ In addition, various studies have shown that peptides and amino acids can be added to traditional ligands to limit the perovskite growth rate and provide greater control over crystal formation.^42–45^ To the best of our knowledge, only one study to date has utilized proteins as the stabilizing ligand for the LHP nanoparticles. Aminzare et al. compared the use of off the shelf proteins (casein, BSA, hemoglobin, lysozyme, trypsin, and pepsin) as ligands for MAPbBr_3_ nanoparticles with small irregular shapes, but with no synthetic control over their resulting dimensions.^46^

Other strategies to stabilize perovskite nanoparticles in polar environments are (1) zwitterionic ligands, which can improve stability by strengthening surface binding and reducing ligand detachment. But it is not perfect, and surface binding can still be disrupted in strong polar solvents.^33–35^ (2) Polymer encapsulation, which enhances stability by physically isolating the nanoparticles from the surrounding environment,^47–49^ and (3) The growth of an inorganic shell, which can enhance stability by adding an inorganic protective layer around the perovskite.^50–52^ These strategies can be effective but often require additional processing steps or more synthetically demanding procedures, and in some cases can compromise nanoparticle integrity or change its properties altogether.^51–54^ Alternatively, self-assembled protein structures that can provide multivalent surface interactions, steric stabilization, and amphiphilicity could be used to stabilize perovskite nanoparticles. This approach could improve the perovskite’s compatibility in polar solvents while maintaining a simple one step synthesis process.

In this study, we were motivated by the bioengineering flexibility the SP1 brings and selected it as our stabilizing protein. SP1 is known for its remarkable stability under extreme conditions, forms a 12-mer self-assembled ring-shaped structure with an inner cavity approximately 2–3 nm in diameter, and has been shown to be used as a template for the synthesis of various inorganic nanomaterials, including CdS,^55^ palladium,^56,57^ and gold nanoparticles.^58^ On the bioengineering front, introducing metal-binding peptides into the protein’s inner pore enhances selectivity for specific reagents. Therefore, we hypothesize that by selecting a tailored design SP1 variant, we can form synergistic perovskite-protein hybrids. Furthermore, the high stability of SP1’s self-assembled structure in organic solvents has been reported,^59^ making them potential candidates as stabilizing ligands to perovskite nanoparticles, an experiment that has not been reported to date. The engineering flexibility of the SP1 can also be used to attach, for example, redox or catalytically active organic moieties that induce charge separation with the perovskite nanoparticles and to direct their assembly on selected substrates, making the perovskite-SP1 hybrid a suitable template for photocatalysis and other light-driven applications.^60–65^

Here, we expand the family of colloidal perovskite nanoparticles to include protein-perovskite hybrids. Specifically, we report that nucleation and growth of quantum-confined perovskite nanoplates will occur only in the presence of SP1. We report the conditions that will allow control over the resulting nanoplates’ quantum confinement, effectively tuning their optical properties. In a systematic study, we show that such control is possible by adjusting the solvent-to-antisolvent ratio, and more surprisingly, also by bioengineering the SP1 variant. In fact, by preselecting the bioengineered variant protein, one will determine the degree of quantum confinement of the resulting perovskite nanoparticles.

In this study, we developed a synthesis for colloidal quantum-confined CsPbBr_3_ perovskite nanoplates stabilized solely by different variants of the SP1. Figure 1a shows the SP1 12-mer structure, and a zoomed monomer with lysine and arginine marked in red and green, respectively, whose terminal amines can interact with the perovskite surface either through a protonated amine (ionic bond) or an unprotonated amine (Lewis acid–base coordination). In addition, the connection can also be established through a coordination bond between the protein’s carboxylic acid functional groups and Pb^2+^ sites.^37,39,66,67^ The three stars in Figure 1a mark the insertion site of the metal-binding peptide added to the SP1, and Table 1 lists the peptide sequences and their acidic/basic residues relevant to the perovskite surface attachment.

**Figure 1:**
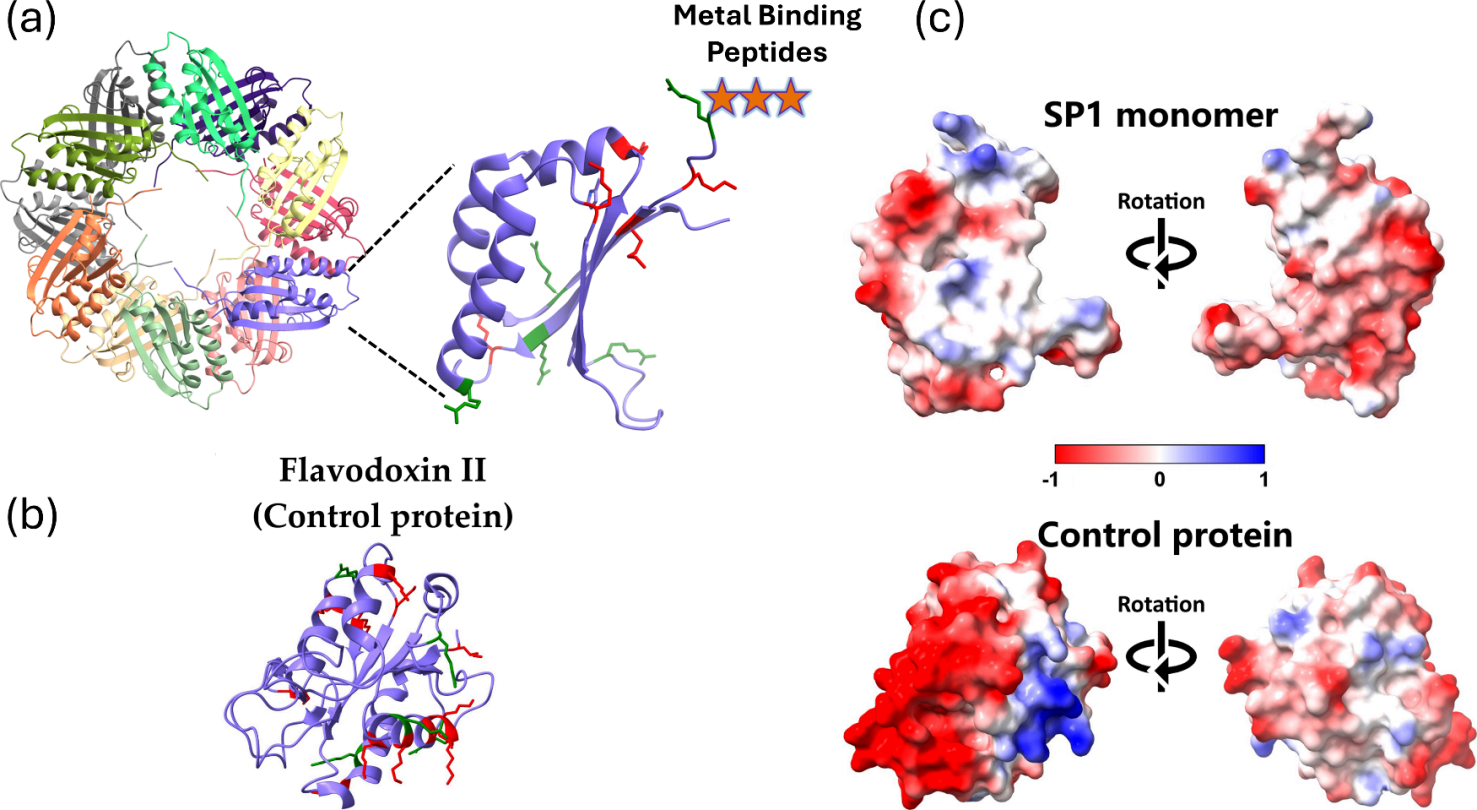
(a) SP1 12-mer ring structure. A zoomed view shows an individual monomer, with lysine residues marked in red and arginine residues marked in green. The SP1 variant is defined by the metal-binding peptide appended at the site marked by three stars. (b) Flavodoxin II (control protein) structure with lysine (red) and arginine (green) highlighted. PDB entry 1YOB. (c) Electrostatic mapping of the SP1 monomer (top) and the control protein (bottom). The colors indicate the negative (red) and positive (blue) charges distributed in the proteins.

**Table 1:**
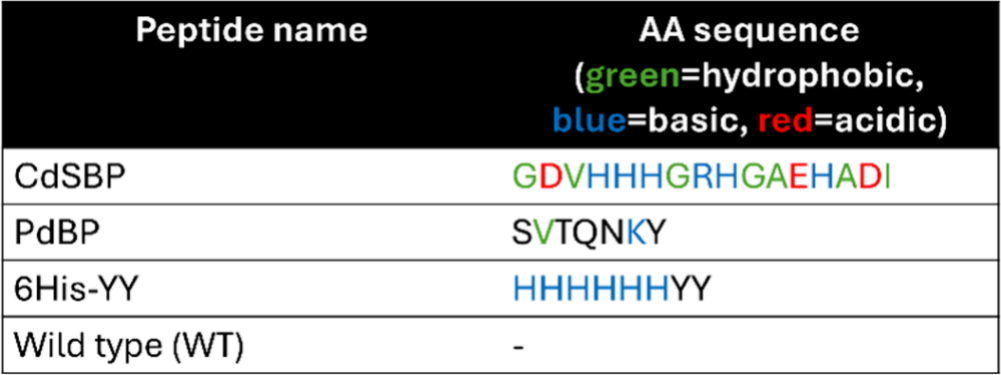
Metal-Binding Peptide of the Different SP1 Variants

To explore how bioengineered SP1 influences the resulting perovskite hybrid nanoplates, we repeated the synthesis with four SP1 variants: CdSBP, PdBP, 6His-YY, and wild type (WT), where CdSBP/PdBP/6His-YY carry peptide additions and the WT is unmodified (Table 1).

Additionally, Flavodoxin II, as shown in Figure 1b, was used as a control protein in the synthesis protocol. As demonstrated in Figure 2, the use of the SP1 protein in the synthesis results in quantum-confined perovskite nanoparticles, whereas using the control protein or omitting the protein altogether results in bulk perovskite (as will be discussed later). The synthesis itself was conducted using the ligand-assisted reprecipitation (LARP) route, as shown in Figure 3a. We first prepared a mother solution by dissolving PbBr_2_ and CsBr in DMSO, then combined an aliquot of this solution with the dried SP1 to generate the “solvent” phase. Toluene served as the antisolvent in the initial synthesis (Figure 3a), obtaining perovskite nanoplates, as shown in the TEM micrograph presented in Figure 3b.

**Figure 2:**
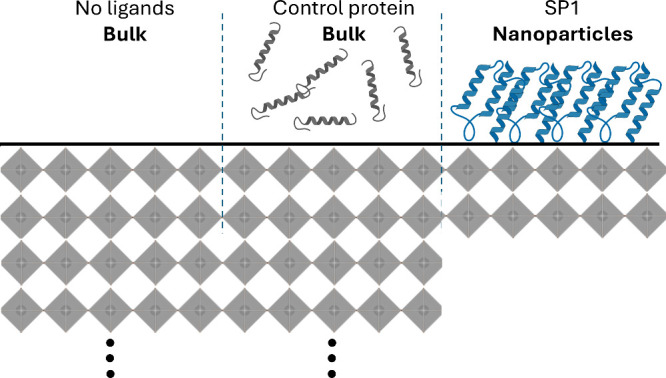
Schematics illustrating the outcomes for no-protein and control-protein conditions versus SP1-added LARP synthesis. Created with BioRender.com.

**Figure 3:**
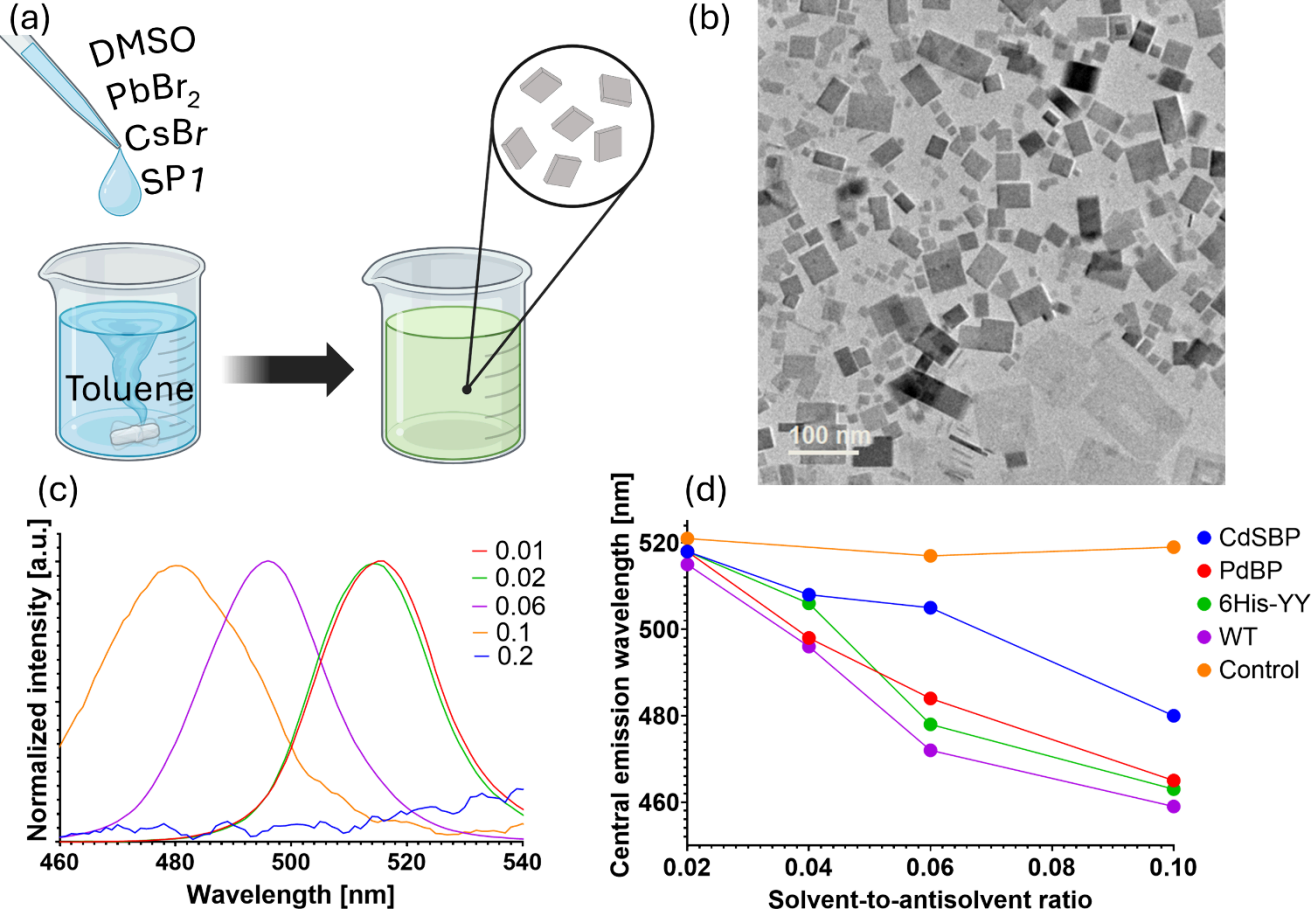
SP1-assisted perovskite synthesis. (a) LARP synthesis scheme describing the formation of SP1-perovskite nanoplates and (b) TEM micrograph of the nanoplates formed. (c) Photoluminescence spectra of the CsPbBr_3_ nanoplates, demonstrating the effect of varying the solvent-to-antisolvent volume ratio at a fixed Pb: SP1 ratio, and (d) Central emission wavelength versus solvent-to-antisolvent ratio of different SP1 variant-perovskite samples. Panel a was created with BioRender.com.

Figure 3c shows how the solvent-to-antisolvent ratio (a key LARP parameter) influences the synthesis. We observe a nonmonotonic trend, where the emission initially blue-shifts and then quenches when the solvent-to-antisolvent ratio exceeds a threshold. This behavior is consistent with insufficient supersaturation to nucleate the perovskite in the LARP route. However, before the quenching threshold, the blue shift is counterintuitive for LARP. Theoretically, increasing the solvent fraction should lower supersaturation, yield fewer nuclei, enable more growth, and thus produce larger particles (a red shift).^68,69^ We hypothesize that SP1 mediates this behavior. As the solvent fraction increases, the amount of SP1 (and dissolved salts) in the synthesis increases, leading to lower supersaturation and fewer (and larger) nuclei. The resulting higher SP1 per nucleus ratio strengthens surface passivation and more effectively limits growth, yielding smaller particles (blue-shift) despite the reduced supersaturation.

To test this hypothesis, we varied the Pb: SP1 ratio (Figure S1 and S2). Figure S1 summarizes the results at two solvent-to-antisolvent ratios. Figure S1a shows that the Pb:SP1 ratio has a minor effect when the solvent-to-antisolvent ratio is low, with minimal spectral shifts observed across the different ratios. We also note that a higher SP1 to particle ratio can have an adverse effect. At higher concentrations of SP1, the sample loses colloidal stability, as SP1 aggregates and precipitates (with minimal changes to the nanoparticles’ optical properties). Optimal colloidal stability is obtained by diluting the “solvent phase” before injecting it into the antisolvent.

In addition, we performed a control synthesis with no protein at all (Figure S3a) and a control protein (flavodoxin II from *Azotobacter vinelandii*, Uniprot accession number P00324), as shown in Figure S3b. We selected this protein as a control due to its comparable dimensions to the SP1 monomer (19 kDa and 13 kDa, respectively). Both control experiments yielded a bulk form of perovskite, as evident in the photoluminescence spectrum shown in Figure S3 and the SEM micrograph in Figure S4. The lack of tunability and the resulting bulk confirm that the preceding effects arise primarily from the specific interaction between SP1 and the perovskite precursors.

Figure 3d plots the central emission wavelength as a function of the solvent-to-antisolvent ratio and the SP1 variant. Across the experimental conditions (Figure 3d and Figures S5–S10), the SP1 variant modulates the synthesis product, with the CdSBP variant exhibiting the smallest spectral shift, and the WT variant has the largest shift. This is in line with the significant difference between the variants, which is their length, where, according to Table 1, the Cd-binding peptide (CdSBP) is the longest. Consistent with this, shorter (or no) inserts correlate with a larger blue shift, indicating more limited thickness growth (Figure S9). We hypothesize that shorter peptides enable tighter SP1 packing at the surface, strengthening passivation and restricting growth. This is illustrated in Table S1, which summarizes the results of the different SP1 variants, along with the approximation of the nanoplate thickness in each case.

Unlike the relatively rigid SP1 backbone, the structure of the bioengineered fused peptide is known to be dynamic and possesses high conformational freedom.^64^ These added peptides limit the subunit’s ability to serve as a well-defined structural contour to stabilize the anisotropic growth of the nanoplates. Consistent with this concept, colloidal nanocrystal studies show that decreasing the effective ligand shell length, for example, by increasing the fraction of shorter ligands within a mixed ligand shell, can promote more anisotropic growth outcomes.^70,71^ In addition, ligands with higher conformational freedom can resist dense, ordered packing at the surface, which can weaken facet-selective stabilization and reduce the precision of anisotropic growth control.^72,73^ Moreover, the amino acid composition of the fused peptide could further affect the protein’s ability to stabilize the structures. While the synthesis is conducted in a nonaqueous medium, which often does not favor stabilization of ionic amino acid side chains, residual hydration or trace water content could stabilize charged residues and dramatically affect the protein behavior in organic solvents.^74^ In this system, positively and negatively charged amino acids may interact with polar perovskite components (for example, Cs^+^, Pb^2+^, and Br^–^), either during the early stages of nucleation as precursor associated species or later during growth when these ions are exposed on the surface of existing nanoplates. These electrostatic interactions can play a critical role in directing nanoplate nucleation, orientation, and anisotropic growth.^75^

As discussed above, the same synthesis protocol was conducted using a control protein, Flavodoxin II (Figure 1b), instead of SP1. Despite Flavodoxin II possessing lysine and arginine residues (highlighted in red and green, respectively) that could bind to the perovskite surface, the synthesis yielded only bulk perovskite (Figure S3b and Figure 3d, control). We attribute this to insufficient structural stability of the control protein in the synthesis environment required for the perovskite synthesis. In addition, Figure 1c displays the electrostatic mapping of both the SP1 monomer and the control protein, with positive charges represented in blue (corresponding to the areas where lysine and arginine residues are located) and negative charges in red. We can see that both proteins are amphiphilic, however, SP1 has more hydrophobic areas, which may support its stability in organic solvents. In addition, when comparing the distribution of negative and positive charges in each protein, we notice that the control protein is more polarized than the SP1. This may help the SP1 perform better as a zwitterionic ligand on the perovskite surface. These observations highlight the robustness of SP1, which enables exceptional stability, even in organic solvents, and underscores the importance of selecting an appropriate protein to match the working conditions and desired final product.

In addition, we verified the perovskite structure by performing X-ray diffraction (XRD) and selected area electron diffraction (SAED) on the nanoparticles formed. Figure S11 presents XRD diffractograms of the perovskite nanoplates produced. The orthorhombic phase matches the expected phase in syntheses conducted at room temperature.^5^ We also confirmed the perovskite structure through SAED, as shown in Figure S12.

Figure 4a shows the photoluminescence spectra of the formed particles after the fine-tuned synthesis. Analysis confirms the nanoplate morphology, and the series-dependent blue shift is consistent with a decrease in plate thickness. The observed blue shift is consistent with quantum confinement along the thickness direction of the CsPbBr_3_ nanoplates. Within the effective mass approximation, the lowest excitonic transition in a semiconductor quantum well increases in energy approximately as 1/L^2^, as the well thickness L approaches the exciton Bohr diameter.^76,77^ This thickness dependent blue shift has been observed experimentally for CsPbBr_3_ nanoplates when their thickness is reduced to a few unit cells.^4,68^ Stacked nanoplates, which are visible in Figure 4b–e in both the XRD diffractogram and the TEM micrograph, enable direct thickness measurements. In small-angle XRD, periodic peaks arising from the stacked structure allow the determination of the thickness using Bragg’s law. Accordingly, we estimate the nanoplate thickness, including two SP1 monomer layers (one on each face), to be 3.69 nm (detailed in SI). This value is smaller than expected, likely due to partial interdigitation of the two protein layers,^78^ however, it still places the plates within the quantum-confined regime. We can also calculate the plate thickness and spacing by using the stacked plates presented in the TEM micrograph in Figure 4c. Applying a Fast Fourier Transform (FFT) mask to the zoomed region marked in Figure 4c (presented in Figure 4d) sharpens the stacking periodicity, yielding a thickness of 3.1 ± 0.2 nm for this sample. Both measurements indicate that the Z-dimension (plate thickness) lies in the quantum-confinement regime, given the Bohr diameter of CsPbBr_3_ is ∼7 nm.^5^

**Figure 4:**
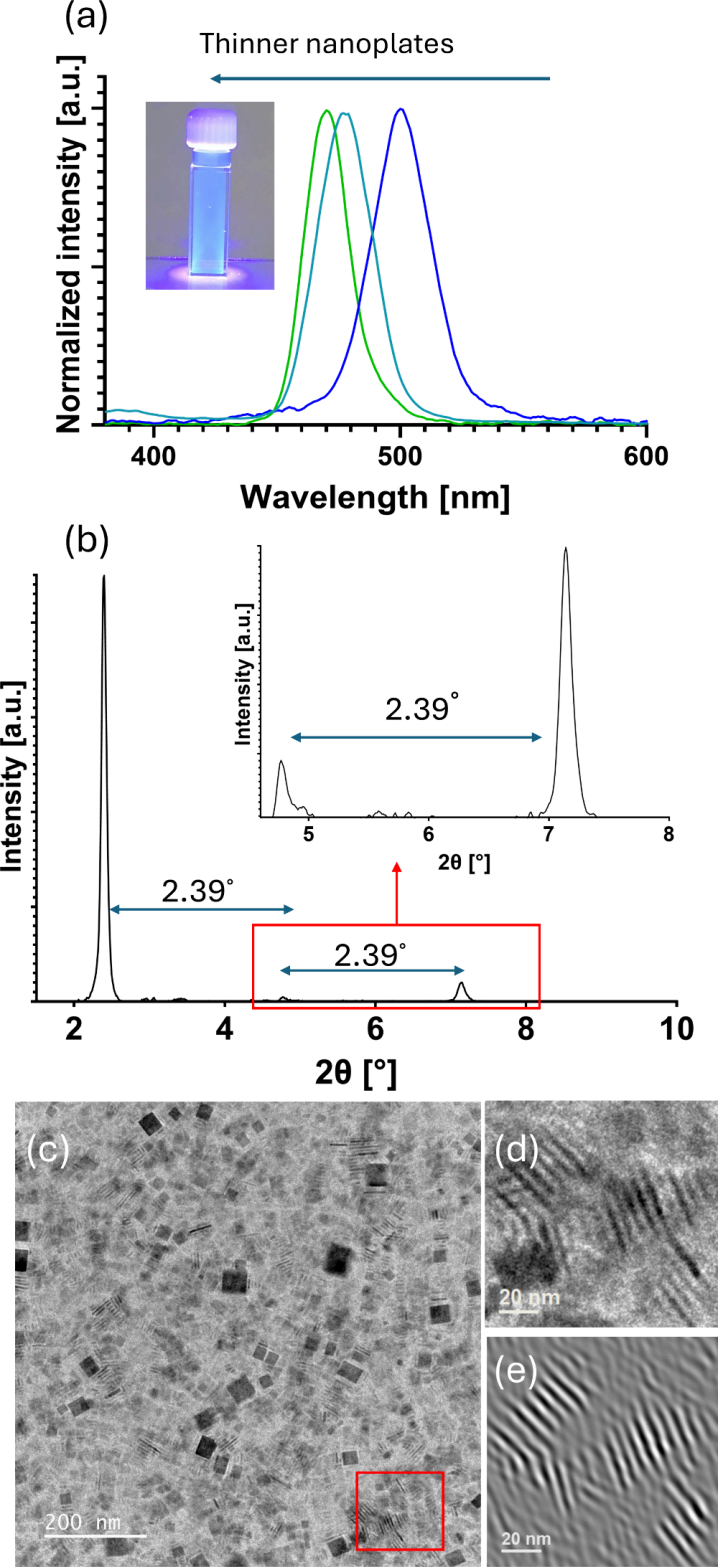
Thickness analysis of SP1-stabilized nanoplates. (a) Photoluminescence spectra for fine-tuned syntheses using the CdSBP variant. (b) Small-angle XRD pattern of stacked nanoplates showing periodic peaks (with 2.39° spacing) of layered structure (inset: blowup of the red-boxed region). (c) TEM micrograph of stacked plates. (d) Blowup of the red-boxed area in (c). (e) FFT-masked view of (d), highlighting the stacking periodicity.

The calculated dimensions of the perovskite nanoplates are larger than the SP1′s 2–3 nm inner cavity, meaning an in-pore template is impossible. This finding indicates that the ring-shaped complex is not responsible for the observed colloidal stabilization. However, since no other ligands are present, the SP1 likely stabilizes the perovskite nanoplates in a different manner. To investigate this, we performed sodium dodecyl sulfate-polyacrylamide gel electrophoresis (SDS-PAGE) to determine the molecular weight of the SP1 and its postsynthesis oligomeric state. Figure S13b shows that the molecular weight of SP1 in the colloidal sample matches the monomeric form of the protein. To study the effect of DMSO on SP1 oligomerization, we tested different SP1 variants (CdSBP and 6His-YY) in different DMSO-water mixtures and monitored their oligomeric state using SDS-PAGE analysis. As shown in Figure S13a, in the absence of DMSO, the hexameric state of each variant could be observed. However, in both 65% and 100% DMSO solutions, the protein hexamers decompose to monomers. These results support the assumption that the free SP1 monomers serve as the stabilizing ligand for the perovskite nanoplates. Moreover, the CdSBP showed a smear in the gel in the presence of DMSO, whereas no such smear was observed for the 6His-YY variant. The appearance of a smear typically indicates reduced sample stability, suggesting that the 6His-YY variant maintains higher structural integrity than CdSBP under these conditions. These observations are consistent with the perovskite synthesis results, which were less efficient when using CdSBP compared with the 6His-YY variant.

To improve SP1 stability during synthesis, we modified the “solvent phase” to a 50:50 (v/v) DMSO: water mixture. Because this “solvent phase” is immiscible with toluene, we used acetone as the antisolvent, which is miscible with both DMSO and water yet a poor solvent for the perovskite, thereby enabling rapid supersaturation and nucleation of nanoplates.^4,79^ The experiments were performed using the most stable variant of SP1, 6His-YY. We achieved optical tunability by varying the solvent-to-antisolvent ratio (Figure 5a), and as achieved in the first version of the synthesis, the nanoparticles maintained their plate form (Figure 5c and Figure S14), likely due to stabilization by SP1 monomers. As shown in Figure 5d, the nanoplates exhibit lattice fringes with a spacing of 0.85 nm, matching the (100) plane of the orthorhombic structure. Using this modified synthesis route, we were able to stabilize the nanoparticles in a polar solvent (isopropanol). The particles remained stable for at least 3 months in isopropanol, as indicated by the aging measurements of the emission in Figure 5b. In addition, TEM micrographs and photos of fresh and aged samples are presented in Figures S15 and S16. The same protocol (i.e., using DMSO: water and acetone) was also used without any protein, resulting in the formation of bulk perovskite, as shown in the photoluminescence spectra in Figure S17a. Moreover, using this synthesis route, the protein-stabilized nanoplates were not stable in their colloidal form when immersed in a nonpolar solvent (hexane and toluene). We assume that this is due to the change made in the synthesis, which maintained the structural stability of SP1 better and thus made it harder to redisperse the final product in a nonpolar solvent compared to a polar one. To validate this, we transformed nanoparticles made in the first version of the synthesis (dispersed in toluene) into isopropanol, which resulted in bulk perovskite, as presented in Figure S17b-c.

**Figure 5:**
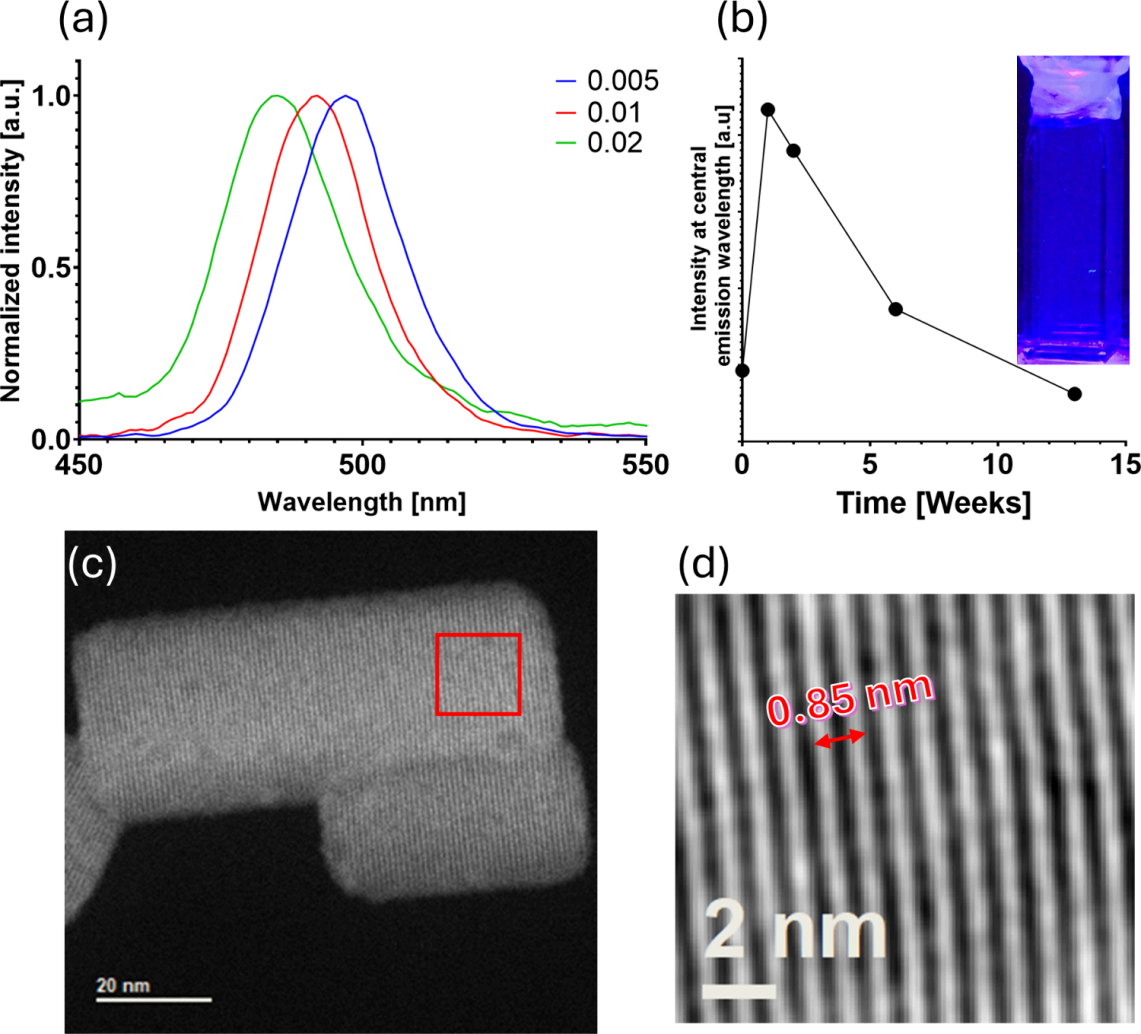
(a) Photoluminescence spectra for the modified synthesis at different solvent-to-antisolvent volume ratios. (b) Aging of the 0.01 solvent-to-antisolvent ratio sample in isopropanol showing the evolution of the PL intensity over time. (c) HRSTEM micrograph of nanoplates dispersed in isopropanol, and (d) is a blowup to the area marked in red in (c) after an FFT mask, showing lattice fringes that match the (100) plane.

To compare the two synthesis routes, we performed time-resolved PL (TRPL), summarized in Figure S18 and Table S2. Figure S18a and c show the spectra of the first version of the synthesis, with varying solvent-to-antisolvent ratios, and Figure S18b and d present the lifetime measurement of the second synthesis route (50/50 DMSO/water), measured after dispersing the nanoplates in isopropanol. Table S2 summarizes the lifetime results of all the samples presented in Figure S18, showing an expected trend in the first synthesis route, as the plate thickness decreases (solvent-to-antisolvent increases in this case), the quantum confinement increases, and the lifetime shortens. We will note that several of these samples require multiexponential fits, which are consistent with thickness polydispersity, a typical characteristic of LARP syntheses. In addition, in an ideal, well-passivated nanoplate population, the TRPL can approach monoexponential decay, reflecting intrinsic band-edge recombination within the nanoplates. In practice, the large surface-to-volume ratio of nanoplates introduces additional surface-related recombination pathways, which typically appear as faster components and shorten the apparent lifetime. Defect or trap assisted processes can add further decay components, contributing to the multiexponential behavior.^80–82^ In contrast, in the second route we detect, in addition to the nanoplates’ band-edge lifetime, sub-30 ps components. The shorter lifetimes can be attributed to the lack of surface passivation of the nanoplates, which can lead to nonradiative trap states.^82–84^ Furthermore, this phenomenon is observed when a polar solvent, such as alcohol, is added to the colloidal perovskite sample, weakening the ligand binding to the surface and thereby decreasing the passivation.^85^

For future steps, as mentioned, perovskites have high photoluminescence quantum yields. Therefore, utilizing these tunable protein-perovskite hybrid photosensitizers for photocatalytic processes may advance the field above the state of the art. It has been shown that perovskites can facilitate light-induced proton or CO_2_ reduction processes for the generation of H_2_ or CH_4_, respectively.^86–88^ It was further presented that, by coupling perovskites with water reduction and water oxidation cocatalysts, full water splitting can be achieved with efficiencies exceeding 2%.^89^ These photocatalytic processes were performed in polar solvents with added water as a proton source. The great and straightforward tunability of the SP1 subunits opens a new route for hybridization to additional catalysts, electrodes, or the formation of spatial separation architecture, which could lead to efficient charge separation. Similar concepts have been demonstrated for other engineered proteins that template, for example, Cd chalcogenide nanocrystals with controlled size and emission, supporting the broader potential of protein scaffolds to tune hybrid photocatalysts.^90–92^ Furthermore, tuning the interfaced amino acid at the perovskite surface or at the solvent surface may lead to improved tunability of the band edges and further control of the required overpotential. The peptides can serve as cocatalysts and provide the necessary environment to facilitate the photooxidation or reduction processes. Light-driven hybrid architectures already enable advanced photonic functions, such as ultrasensitive surface-enhanced Raman scattering (SERS) detection of water contaminants and chiroptical responses in chiral tin bromide crystals,^93,94^ indicating that bioengineered SP1 perovskite nanoplates may provide a platform to explore related sensing and chiral photonic designs.

To conclude, we successfully synthesized CsPbBr_3_ perovskite nanoplates that are passivated solely by the monomeric form of the SP1. By bioengineering the SP1 variants, we directly control the resulting nanoplate thickness and, consequently, the exciton’s quantum confinement as observed by the emission wavelength. In addition, the amphiphilic nature of SP1 stabilizes the perovskite nanoplates in a polar environment, advancing the goal of implementing perovskite nanoparticles in aqueous catalytic applications one step further. The vision is to use the flexibility of SP1 to engineer protein-perovskite hybrids with specific functionalities. Future follow-up studies could leverage this versatility and incorporate redox-active or catalytic peptides, thereby enabling effective charge transfer from the perovskite nanoplates to the attached moiety. We thus signal that protein-perovskite hybrids may soon join the extended family of next-generation catalytic materials.

## Supplementary Material



## Notes

Materials, synthesis, and methods of characterization, additional absorption and emission spectra, SEM, TEM, SAED, XRD, SDS-PAGE, and TRPL (PDF)
