# Supplementary material for: Bioengineered Protein Stabilized Perovskite Nanoplates in Polar Solvents
Source: Nano Lett. 2026 Mar 8;26(10):3596–605. doi: 10.1021/acs.nanolett.6c00282 (PMC13003495; doi:10.1021/acs.nanolett.6c00282)
Supplement: Supplementary file 1 [file nl6c00282_si_001.pdf]

Supporting information

Bioengineered Protein Stabilized Perovskite Nanoplates in Polar Solvents

Emma H. Massasa[1], Oren Bachar[2], Arad Lang[1], Omer Yehezkeli[2,3] and Yehonadav Bekenstein[1,3,4*]

(1) Department of Materials Science and Engineering, Technion - Israel Institute of Technology, 3200003 Haifa, Israel

(2) Faculty of Biotechnology and Food Engineering, Technion - Israel Institute of Technology, 3200003 Haifa, Israel

(3) The Resnick Sustainability Center for Catalysis, Technion - Israel Institute of Technology, 3200003 Haifa, Israel

(4) The Solid-State Institute, Technion - Israel Institute of Technology, 3200003 Haifa, Israel

METHODS

Materials

Lead bromide ($PbBr_2$, 99.999% Sigma Aldrich), Cesium bromide (CsBr, 99%, AA Blocks), dimethyl sulfoxide (DMSO, 99.5%, Sigma Aldrich), water HPLC grade (Sigma Aldrich), toluene (AR, Bio Laboratories), acetone (AR, Bio Laboratories), and isopropanol (AR, Bio Laboratories).

Protein purification and preparation:

The purification of WT-SP1, CdSBP-SP1, and PdBP-SP1, as well as flavodoxin II, was performed by anion exchange chromatography followed by size exclusion, as previously reported.[1] YY6His-SP1 was purified using affinity chromatography as reported in our earlier studies.[2] The purity of the isolated proteins was characterized using SDS-PAGE analysis (SurePAGE, Bis-Tris 4-20%, GenScript). Purified proteins were quantified using UV-Vis spectroscopy at 280 nm. Then, a fixed amount of purified protein was freeze-dried overnight using a lyophilizer, resulting in a dry white powder to be used for perovskite synthesis.

Synthesis:

Based on previous studies,[3,4] the preparation of the precursor solution was performed as follows: for the first version of the synthesis, a mixture of 73 mg of $PbBr_2$ and 43 mg of CsBr was dissolved in 5 ml of dimethyl sulfoxide (DMSO) and stirred for 2 hours. In the modified synthesis version, first, the salts are dissolved in 2.5 ml of DMSO, and then 2.5 ml of deionized water is added.

Next, 3 mg of freeze-dried SP1 is dissolved in 1-2 ml of the prepared precursor solution. If the nanoparticle sample is not colloidally stable after preparation, the precursor solution can be diluted (2X or 3X) before injecting it into toluene.

Then, a specified volume of the protein-precursor solution is injected into 5 ml of toluene (or acetone) under constant stirring. For the modified synthesis version, the sample is then centrifuged at 7500 rpm for 5 min and re-dispersed in isopropanol.

Characterization

Transmission Electron Microscopy (TEM)

Samples for TEM were prepared by drop-casting the colloidal dispersion onto carbon-coated copper grids at room temperature. Imaging was performed on a FEI Tecnai G2 T20 S-Twin TEM operated at 200 kV. The HR-STEM micrograph was achieved on a high-resolution transmission electron microscope FEI Titan 80–300 kV FEG-S/TEM using 200 KeV acceleration voltage.

Scanning Electron Microscopy (SEM)

Samples for the SEM were prepared by drop casting the solution onto silicon substrates at room temperature. Imaging was performed on a Zeiss Ultra-Plus FEG-SEM. Samples were placed at a 4 mm working distance and measured with an acceleration voltage of 2 kV.

X-ray Diffraction (XRD)

Samples were prepared by drop-casting the solution onto glass slides. X-ray diffraction was collected on a Rigaku SmartLab (9 kW) using Cu Kα radiation (λ = 1.5406 Å). Patterns were recorded over 2θ = 1–70°, capturing low-angle reflections from the nanoplate stacking and the perovskite peaks across both small- and wide-angle regimes.

Sodium dodecyl sulfate–polyacrylamide gel electrophoresis (SDS-PAGE)

After synthesis, the samples were directly loaded into the gel cassette without the addition of an SDS-PAGE sample buffer. To prevent sample flotation resulting from density differences, the level of the running buffer was initially maintained below the height of the wells during the first 10 minutes of electrophoresis. Following this migration period, the running buffer was then filled to cover the gel cassette as per standard procedure.

Optical Characterizations

Spectra of the colloidal solutions were measured in a quartz cuvette using a Biotek Synergy H1 instrument equipped with a xenon lamp (Xe900). Photoluminescence spectra were acquired by illuminating the samples at 350 nm. Time-resolved photoluminescence (TRPL) measurements were performed on an Edinburgh FLS1000 photoluminescence spectrometer, using excitation at 375 nm with a pulsed diode laser, at room temperature. The samples were measured in a quartz cuvette.

In addition, photoluminescence quantum yield (PLQY) was performed in an Edinburgh FLS1000 instrument using a cuvette and an integrated sphere holder. However, the results were below 1%. We hypothesize that this is due to the tendency of the protein to self-assemble, and we observe this tendency in our SP1-capped nanoplate samples, leading to larger protein assemblies that are likely larger than the optical wavelength used and therefore contribute strong elastic scattering. In this situation, even small differences in scattering between the sample and the corresponding reference measurement can influence subtraction-based absolute PLQY evaluation in an integrating sphere and lead to a low apparent PLQY.

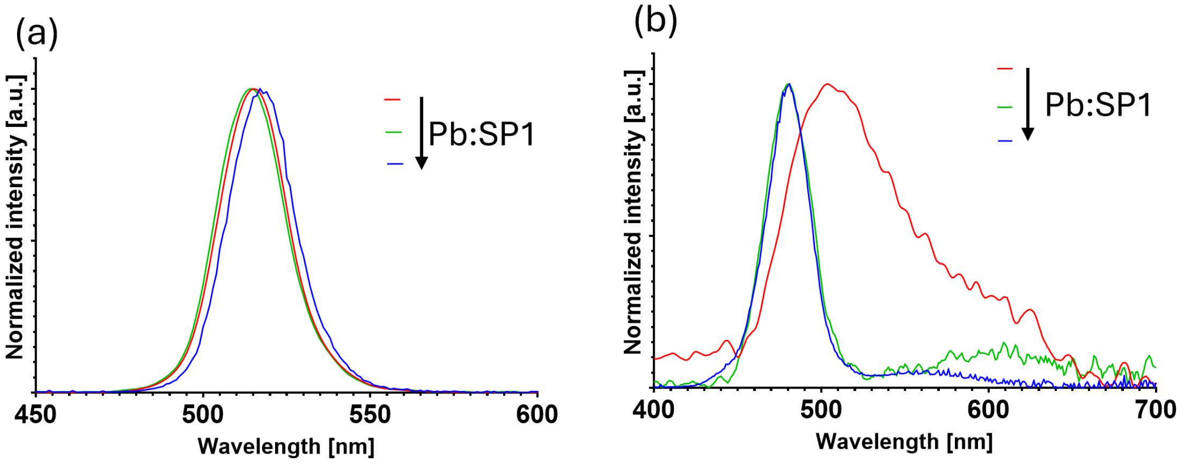

Figure S1. Changing synthesis parameters. Photoluminescence spectra of (a) decreasing Pb: SP1 molar ratios with a 0.02 solvent-to-antisolvent ratio. (b) decreasing Pb: SP1 molar ratios with a 0.1 solvent-to-antisolvent ratio.

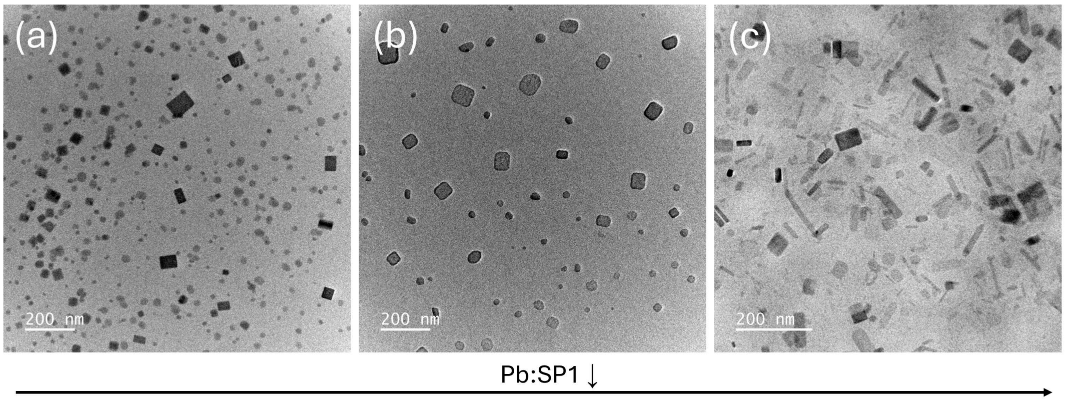

Figure S2. TEM micrographs of CdSBP-capped nanoplates using a 0.1 solvent-to-antisolvent ratio, with different Pb: SP1 ratios

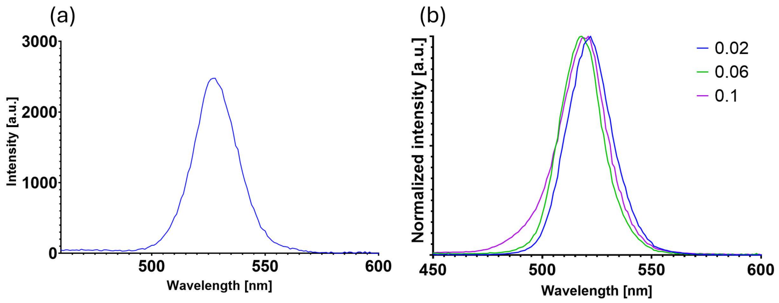

Figure S3. Photoluminescence spectra of (a) a control experiment with a 0.1 solvent-to-antisolvent ratio containing no protein, and (b) a control with a non-SP1 protein, with varying ratios of solvent-to-antisolvent

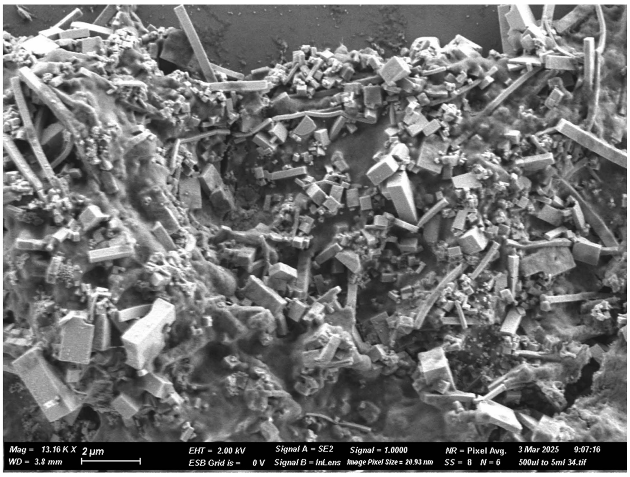

Figure S4. SEM micrograph of the bulk perovskite sample

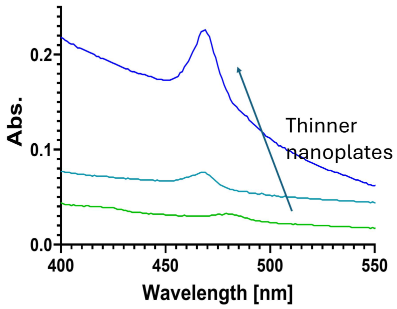

Figure S5. Absorption spectra of the fine-tuned nanoplate samples

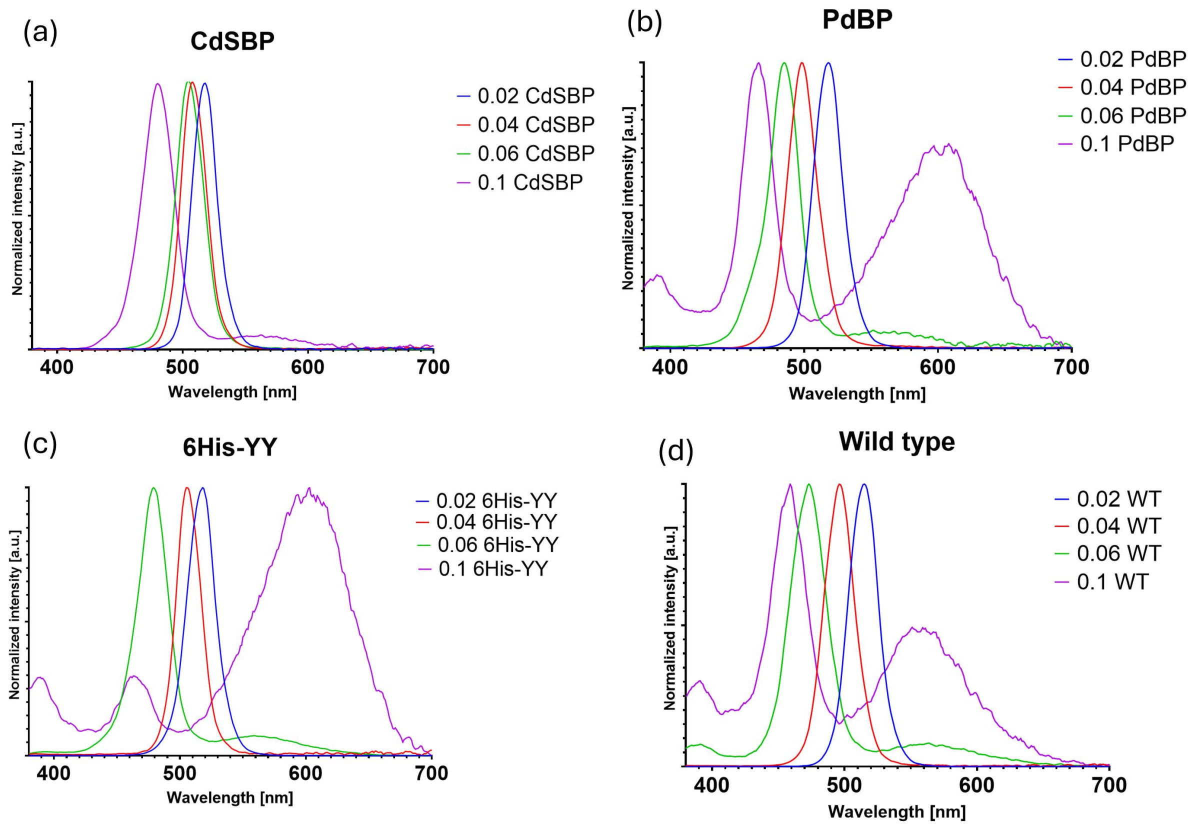

Figure S6. Normalized photoluminescence dependence on solvent volumes with different SP1 variants. (a) CdSBP, (b) PdBP, (c) 6His-YY, and (d) wild type

We note that when the yield of the synthesis was low, additional peaks appeared in the photoluminescent spectra, as shown in Fig. S6. These peaks can be attributed to the SP1 protein, which is more prominent due to the low emission from the perovskite nanoplates. To illustrate the differences in yields, we also present the non-normalized photoluminescent spectra in Fig. S7. In addition, for a better comparison between the different SP1 variants, the photoluminescence spectra are divided according to the "solvent phase" volumes in Fig. S8.

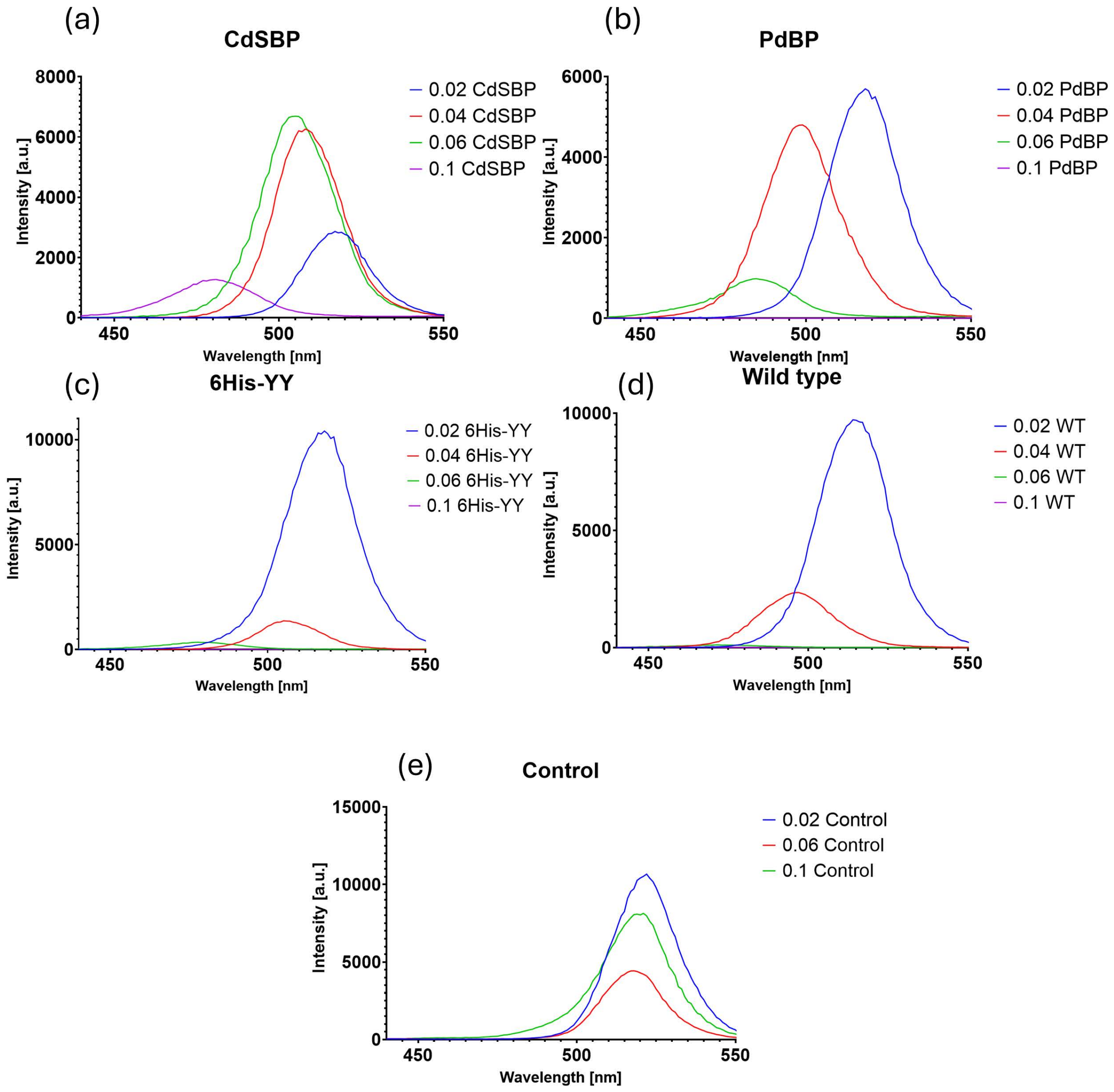

Figure S7. Photoluminescence dependence on solvent volumes with different SP1 variants. (a) CdSBP, (b) PdBP, (c) 6His-YY, (d) wild type, and (e) control protein (non-SP1)

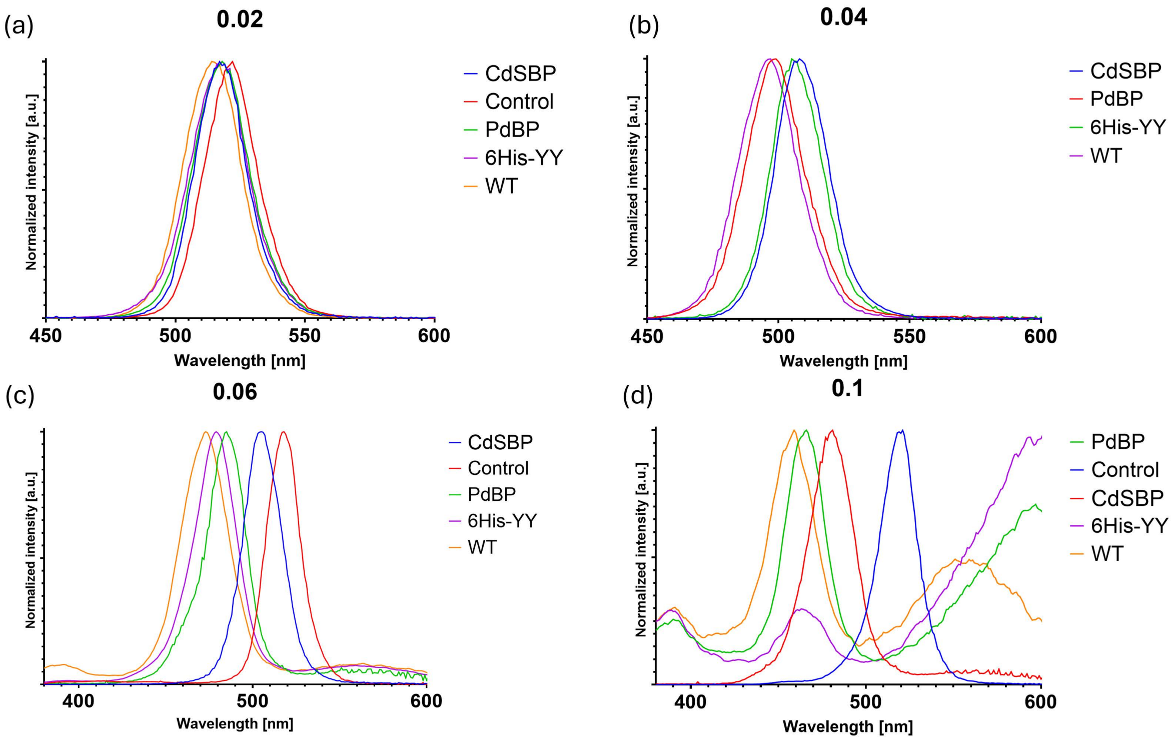

Figure S8. Photoluminescence of samples with different SP1 variants at increasing solvent-to-antisolvent ratios: (a) 0.02, (b) 0.04, (c) 0.06, and (d) 0.1.

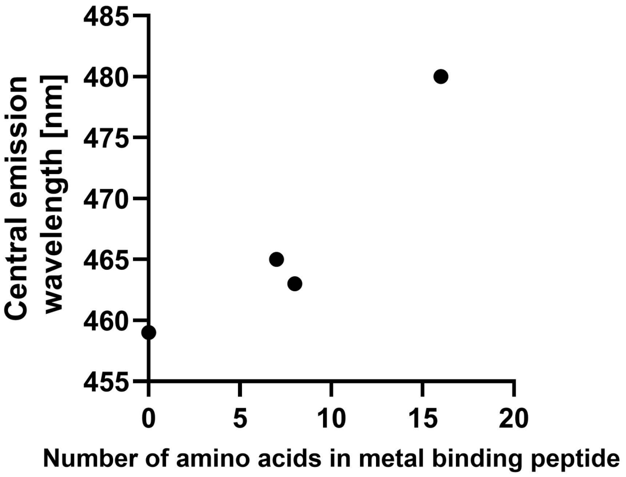

Figure S9. The number of amino acids in the different metal-binding peptides on the SP1 as a function of the most blue-shifted emission central wavelength.

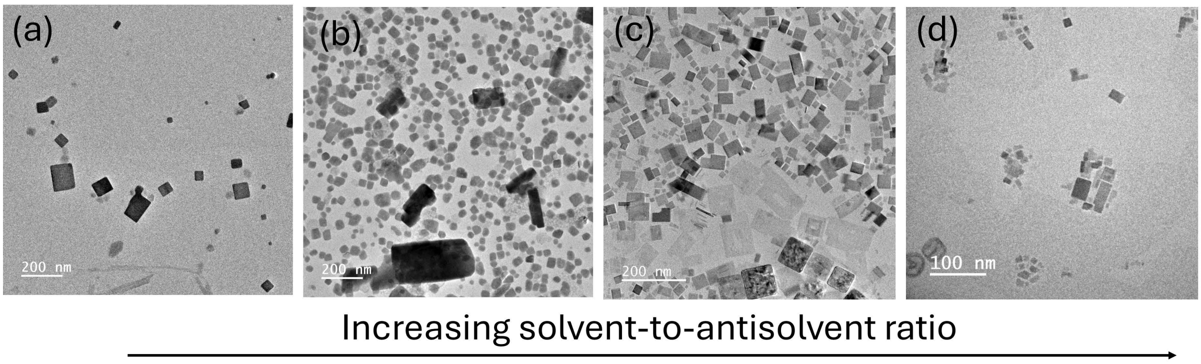

Figure S10. TEM micrographs of CdSBP-capped $CsPbBr_3$ nanoplates made with different solvent-to-antisolvent ratios of (a) 0.02, (b) 0.06, (c) 0.08, and (d) 0.1

To summarize the effect of the different SP1 variants on the resulting nanoplates, we list the central emission wavelength and full width at half maximum (FWHM) in Table S1. From these parameters and based on the work of Bekenstein *et al.*[5] and Akkerman *et al.*[6] We could estimate the monolayer (ML) thickness of each sample.

Table S1. Summary of the effect of the different SP1 variants on nanoplate properties in the 0.06 and 0.1 solvent-to-antisolvent ratios

**0.06**			
SP1 variant	Central emission wavelength [nm]	FWHM [nm]	Approximated plate thickness
CdSBP	505	26	10-11 ML
PdBP	484	28	6-8 ML
6His-YY	478	29	5-7 ML
Wild type	475	34	5-7 ML
**0.1**			
CdSBP	480	30	6-8 ML
PdBP	465	28	5-6 ML
6His-YY	463	25	5-6 ML
Wild type	459	33	4-6 ML

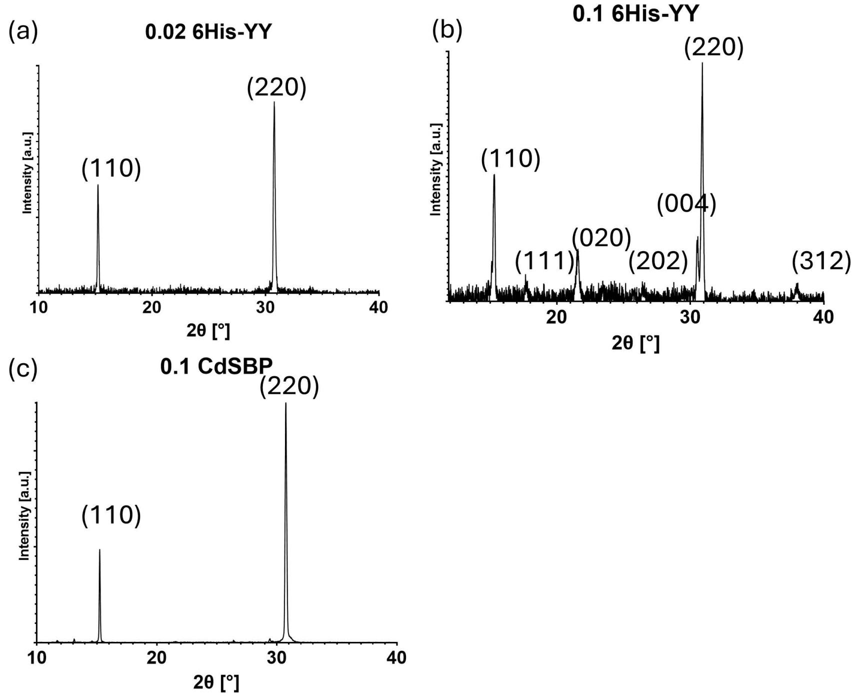

Figure S11. XRD diffractograms of different samples showing a match to orthorhombic $CsPbBr_3$ perovskite, according to JCPDS card 04-014-9676

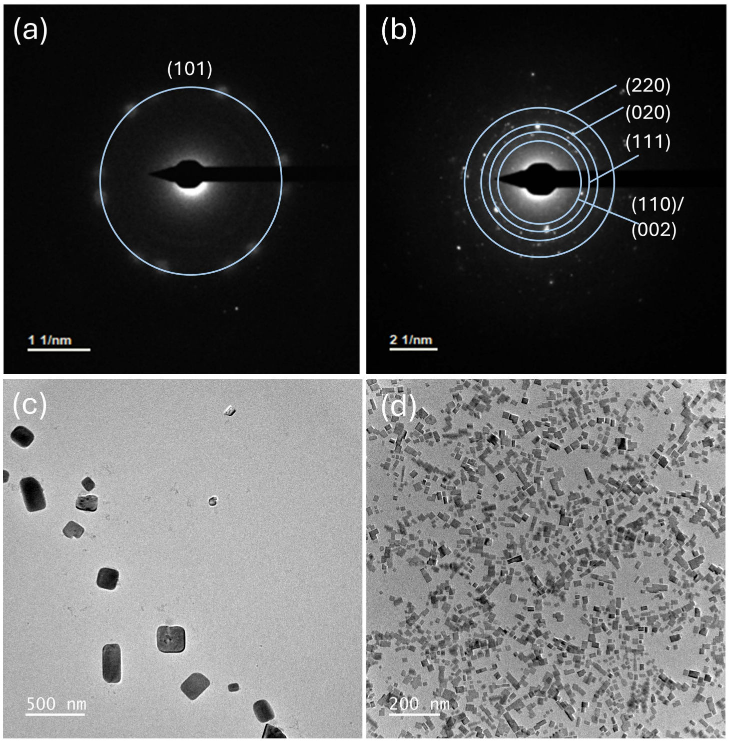

Figure S12. (a) and (b) selected area electron diffraction (SAED) patterns of the nanoplates shown in (c) and (d), respectively, matching the orthorhombic $CsPbBr_3$ phase

Using low angle X-ray diffraction, we estimated the thickness of the nanoplates with two ligand layers (one on each side) from Bragg's law:

$$(1)\ n\lambda = 2d\sin\theta$$

In a stacked assembly of nanoplates, the low-angle reflection corresponds to the periodicity of the stacked structure, where d is the spacing between plate centers, equal to the sum of the nanoplate thickness and the ligand shells. In Fig. 4b, the first-order reflection appears at $2\theta = 2.39°$. Taking Cu Kα radiation ($\lambda = 0.154$ nm) and $n = 1$, we obtain:

$$d = \frac{n\lambda}{2\sin\theta} = \frac{0.154\ nm}{2\sin\frac{2.39}{2}} = 3.69nm$$

This value therefore represents the total thickness of a protein-coated nanoplate (inorganic core plus two ligand layers).

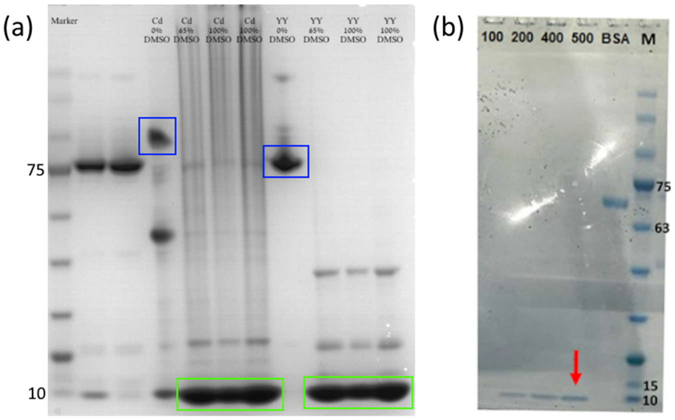

Figure S13. SDS-PAGE analysis to determine SP1's oligomeric state. (a) The effect of DMSO percentage in water on the oligomeric state of the CdSBP (Cd) and the 6His-YY (YY) variants. Blue and green rectangles represent SP1's hexameric and monomeric states, respectively. (b) Post-synthesis SP1-perovskite colloidal sample. The number in each well represents the injection volume of the SP1-precursor solution to the antisolvent. Bovine serum albumin (BSA) was used as a reference protein with a known molecular weight that went through the same conditions before SDS-PAGE analysis. The numbers near the bands of the protein marker indicate their corresponding molecular weight in kDa.

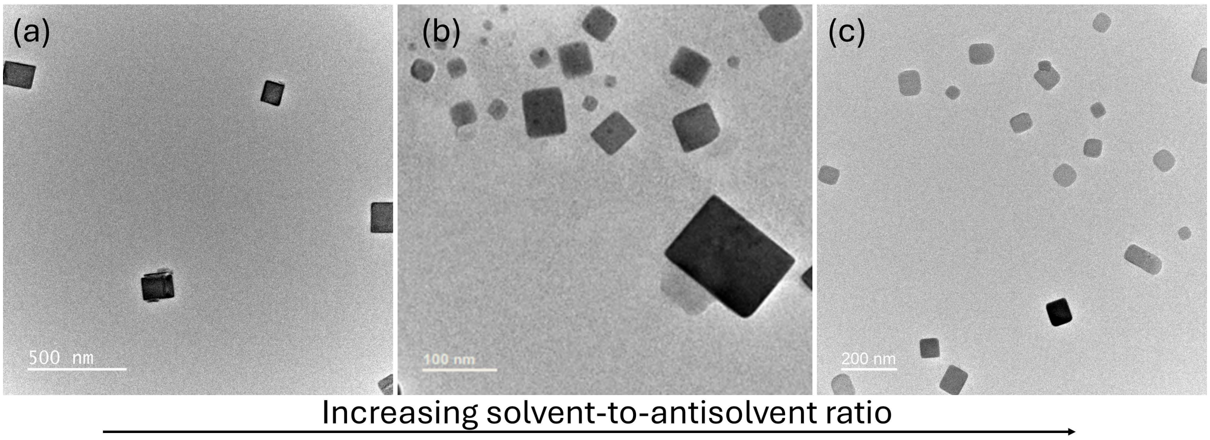

Figure S14. TEM micrographs of 6His-YY -capped $CsPbBr_3$ nanoplates dispersed in isopropanol and made with the modified synthesis using different solvent-to-antisolvent ratios of (a) 0.005, (b) 0.01, and (c) 0.02

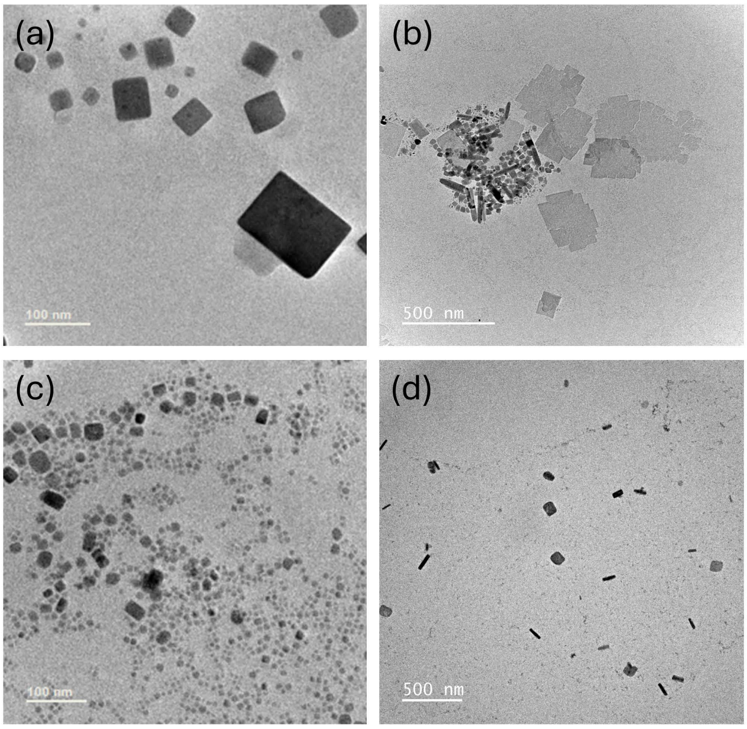

Figure S15. TEM micrographs of fresh and aged samples. (a) and (b) are fresh and 1-month aged 6His-YY capped nanoplates dispersed in isopropanol, respectively. (c) and (d) are fresh and 5-month aged CdSBP capped nanoplates dispersed in toluene, respectively.

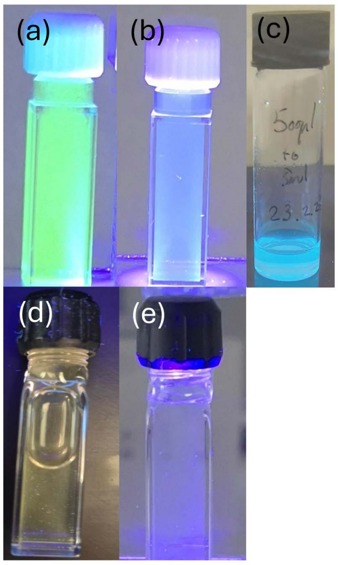

Figure S16. Photos of the different syntheses. Nanoplates capped with CdSBP and dispersed in toluene when using (a) 0.04 and (b) 0.1 solvent-to-antisolvent ratio, and (c) is (b) after 6 months. Nanoplates capped with 6His-YY made via the modified synthesis and dispersed in isopropanol. (d) and (e) are the aged sample before and during illumination, respectively.

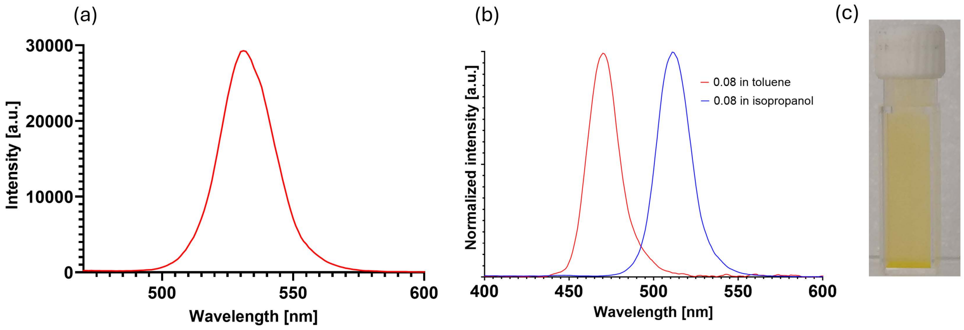

Figure S17. Photoluminescence spectra of (a) a control experiment containing no protein at all in the 50-50 DMSO-water synthesis version, with a 0.01 solvent-to-antisolvent ratio. (b) a sample with a 0.08 solvent-to-antisolvent ratio made through the 1st synthesis version (only DMSO as solvent) in toluene and in isopropanol, showing the lack of stabilization in a polar solvent. (c) An image of the sample in (b) after transforming the particles to isopropanol

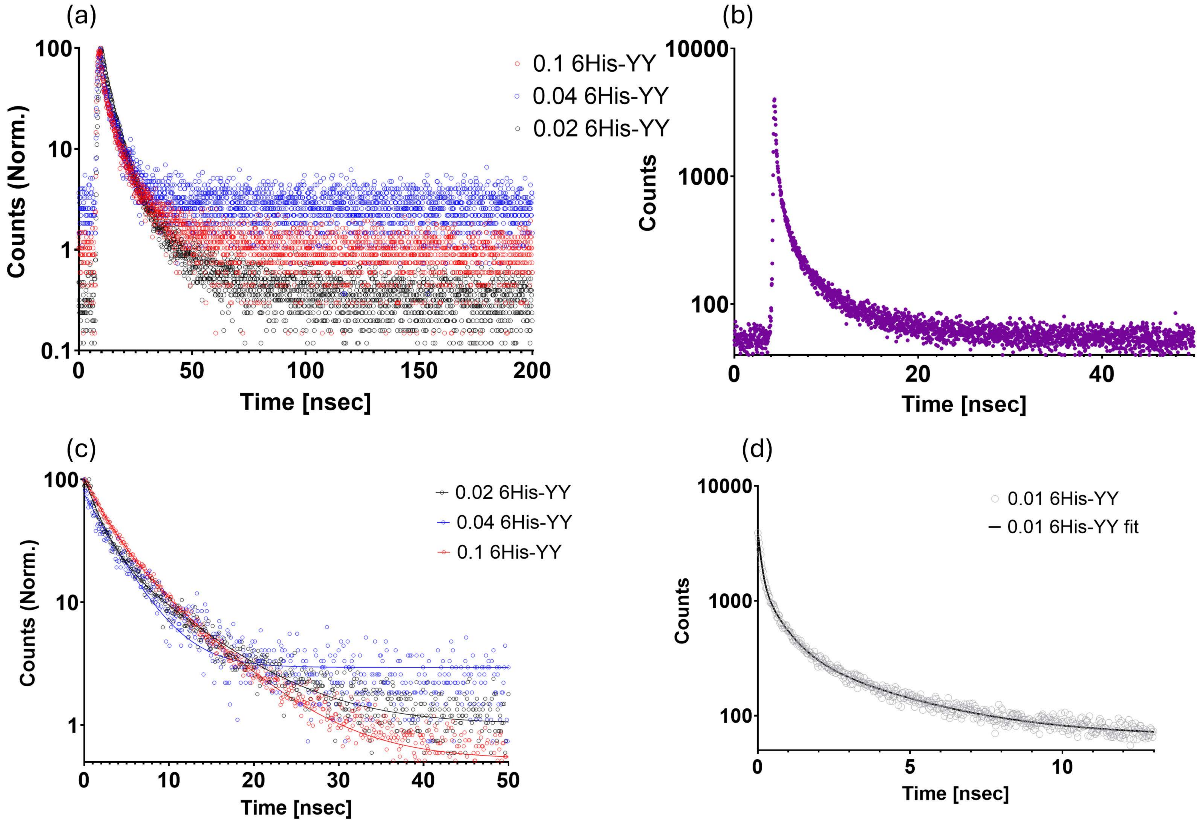

Figure S18. Time-resolved PL of (a) the first synthesis version with the 6His-YY SP1 variant, sampling different solvent-to-antisolvent ratios, and (b) the second synthesis version with a 0.01 solvent-to-antisolvent ratio containing the 6His-YY SP1 variant. (c) and (d) are the result of (a) and (b) with the fitting, respectively.

Table S2. Summarized results of the TRPL measurements

sample	Emission wavelength	LTs
**0.01 6His-YY (in isopropanol)**	490 nm	<30 ps (46%)<br>3.2 ns (54%)
**0.02 6His-YY**	503 nm	3.13 ns (57%)<br>9.39 ns (43%)
**0.04 6His-YY**	495 nm	4.3 ns
**0.1 6His-YY**	470 nm	2.2 ns (39%)<br>9.4 ns (61%)

REFERENCES

(1) Bachar, O.; Meirovich, M. M.; Zeibaq, Y.; Yehezkeli, O. Protein-Mediated Biosynthesis of Semiconductor Nanocrystals for Photocatalytic NAD(P)H Regeneration and Chiral Amine Production. *Angewandte Chemie - International Edition* 2022, *61* (23), 202202457.

(2) Bachar, O.; Meirovich, M. M.; Kurzion, R.; Yehezkeli, O. In Vivo and in Vitro Protein Mediated synthesis of Palladium Nanoparticles for Hydrogenation. *Chemical Communications* 2020, *56* (76), 11211–11214.

(3) Li, X.; Wu, Y.; Zhang, S.; Cai, B.; Gu, Y.; Song, J.; Zeng, H. CsPbX3 Quantum Dots for Lighting and Displays: Room-Temperature Synthesis, Photoluminescence Superiorities, Underlying Origins and White Light-Emitting Diodes. *Adv. Funct. Mater.* 2016, *26* (15), 2435–2445.

(4) Welyab, G.; Abebe, M.; Mani, D.; Thankappan, A.; Thomas, S.; Aga, F. G.; Kim, J. Y. All-Inorganic CsPbBr3 Perovskite Nanocrystals Synthesized with Olive Oil and Oleylamine at Room Temperature. *Micromachines (Basel).* 2023, *14* (7), 1332.

(5) Bekenstein, Y.; Koscher, B. A.; Eaton, S. W.; Yang, P.; Alivisatos, A. P. Highly Luminescent Colloidal Nanoplates of Perovskite Cesium Lead Halide and Their Oriented Assemblies. *J. Am. Chem. Soc.* 2015, *137* (51), 16008–16011.

(6) Akkerman, Q. A.; Motti, S. G.; Srimath Kandada, A. R.; Mosconi, E.; D'Innocenzo, V.; Bertoni, G.; Marras, S.; Kamino, B. A.; Miranda, L.; De Angelis, F.; Petrozza, A.; Prato, M.; Manna, L. Solution Synthesis Approach to Colloidal Cesium Lead Halide Perovskite Nanoplatelets with Monolayer-Level Thickness Control. *J. Am. Chem. Soc.* 2016, *138* (3), 1010–1016.
